# Head-to-Head Comparison of Muscle Energy Technique and Joint Mobilization on Pain Improvement in Mechanical Neck Pain and Related Outcomes: A Systematic Review and Meta-Analysis

**DOI:** 10.3390/healthcare14213719

**Published:** 2026-11-02

**Authors:** Lee-jin Woo, Da-won Ko, Eun-Jung Lee, Sujeong Yeom, Joung Seog Seo, Boram Lee, Eun-Bum Kang

**Affiliations:** 1 College of Korean Medicine, Daejeon University, 62, Daehak-Ro, Dong-Gu, Daejeon 34520, Republic of Korea; origin@edu.dju.ac.kr (L.-j.W.); 20202137@edu.dju.ac.kr (D.-w.K.); 2 Department of Korean Rehabilitation Medicine, College of Korean Medicine, Daejeon University, Daejeon 34520, Republic of Korea; jungkahn@dju.kr (E.-J.L.);; 3 Department of Digital New Technology Convergence, College of Digital Future Convergence, Daejeon University, 62, Daehak-Ro, Dong-Gu, Daejeon 34520, Republic of Korea; 4 KM Science Research Division, Korea Institute of Oriental Medicine, Daejeon 34054, Republic of Korea; 5 Department of Sports Health Rehabilitation, Daejeon University, 62, Daehak-Ro, Dong-Gu, Daejeon 34520, Republic of Korea

**Keywords:** mechanical neck pain, muscle energy technique, joint mobilization, Mulligan mobilization, neck-related disability, meta-analysis

## Abstract

**Background**: Mechanical neck pain (MNP) stems from muscular or articular dysfunction. Muscle energy technique (MET) and joint mobilization (JM) address these mechanical causes; however, comparative evidence remains limited. This systematic review and meta-analysis compared the effect of MET and JM in patients with MNP. **Methods**: Electronic databases (PubMed, Cochrane Library, EMBASE) were searched up to 12 September 2025. Randomized controlled trials (RCTs) directly comparing MET and JM were included following PRISMA 2020 guidelines. Pain intensity was prespecified as the primary outcome, while functional disability and cervical range of motion (ROM) were secondary outcomes. Risk of bias was assessed using the Cochrane RoB 2.0 tool. Random-effects meta-analyses were conducted, with subgroup analyses stratified by JM technique and treatment duration. The certainty of evidence was evaluated using the Grading of Recommendations, Assessment, Development, and Evaluation (GRADE) approach. **Results**: Six RCTs encompassing 381 randomized participants were included. Of these, 301 participants contributed to the pain and disability analyses, and 250 contributed to the flexion and extension ROM analyses. Risk-of-bias assessment indicated that all included studies were judged to have “some concerns,” primarily owing to bias in outcome measurement. Overall pooled analyses showed no significant difference between MET and JM in pain reduction (MD = 1.15; 95% CI −0.26 to 2.57) or disability improvement, despite considerable heterogeneity (*I*^2^ > 90%). In an exploratory subgroup analysis of two trials, point estimates favored JM in studies using a 4-week Mulligan mobilization protocol. **Conclusions**: The pooled analyses found no statistically significant differences between MET and JM in pain, disability, or cervical flexion ROM, whereas extension ROM favored JM. Findings from the two trials of 4-week Mulligan mobilization remain exploratory and do not establish superiority or an optimal treatment duration. **Protocol registration**: PROSPERO CRD420251166998.

## 1. Background

Mechanical neck pain (MNP) is a prevalent musculoskeletal disorder characterized by pain originating from cervical structures in the absence of identifiable neurological pathology [1]. Its multifactorial etiology commonly involves muscular dysfunction, facet joint pathology, and sensorimotor control disturbances [2], which may contribute to abnormal cervical loading and persistent nociceptive input [3].

Globally, neck pain affects up to 70% of individuals during their lifetime [4] and frequently recurs or becomes chronic, with nearly 50% experiencing persistent or recurrent symptoms over the long term [5]. It imposes a substantial public health and economic burden through reduced productivity, impaired quality of life, and increased healthcare utilization [4], highlighting the need for effective management strategies [6].

MNP is commonly managed with medications, physical modalities, manual therapy, and therapeutic exercise according to current clinical guidelines [7]. However, pharmacological interventions, including non-steroidal anti-inflammatory drugs (NSAIDs) and injection-based therapies, typically provide short-term benefits and may lead to adverse effects with prolonged use [8]. Manual therapy and exercise-based interventions, by contrast, directly target the underlying mechanical impairments and improve long-term outcomes [9]. Current evidence accordingly favors multimodal approaches that pair cervical mobilization or manipulation with targeted therapeutic exercise, particularly in patients with mobility deficits [10].

Muscle energy technique (MET) and joint mobilization (JM) are commonly used manual therapies for MNP. MET involves brief isometric muscle contractions and is traditionally explained through post-isometric relaxation (PIR) and reciprocal inhibition (RI) [11]. JM applies graded forces or mobilization with movement to improve cervical mobility and reduce pain [12]. Common JM approaches include Maitland and Cyriax techniques and Mulligan mobilization, including sustained natural apophyseal glides (SNAGs) and natural apophyseal glides (NAGs) [13,14]. These approaches differ in their application and proposed mechanisms, although the physiological mechanisms underlying their clinical effects remain incompletely established [15,16].

Although previous studies have reported that MET and JM can each alleviate pain and improve function, most investigations have evaluated these techniques independently rather than comparatively [17,18]. Despite their widespread clinical application, direct comparisons of MET and JM remain scarce, leaving uncertainty regarding which technique may offer greater clinical advantages for specific patient populations. Therefore, the primary research question of this systematic review and meta-analysis was whether MET and JM differ in their effects on pain intensity in patients with MNP, with neck-related functional disability and cervical range of motion assessed as secondary outcomes, without a prespecified directional hypothesis favoring either intervention.

## 2. Methods

This review was conducted in accordance with the Preferred Reporting Items for Systematic Reviews and Meta-Analyses (PRISMA) 2020 guidelines [19]. The review protocol was registered at the International Prospective Register of Systematic Reviews PROSPERO (Prospero ID: CRD420251166998) on 29 October 2025. No significant deviations from the original protocol occurred during the review process. The study was conducted without prior publication of the study protocol.

### 2.1. Eligibility Criteria

The “Population Intervention Comparison Outcome Study-design” (PICOS) framework was used to establish inclusion and exclusion criteria.

• Participants: Patients with MNP or nonspecific neck pain without neurological deficits were eligible. No restrictions were applied regarding participant age, sex, or duration of symptoms (acute, subacute, or chronic). MNP was interpreted as musculoskeletal neck pain without neurological deficits, encompassing nonspecific neck pain and symptomatic cervical spondylosis, while recognizing differences between these diagnostic categories.
• Interventions: Eligible studies directly compared MET—including PIR or RI—with JM techniques, such as Maitland mobilization, Mulligan mobilization (including sustained or natural apophyseal glides [SNAGs/NAGs]), or Cyriax mobilization. Studies allowing concurrent conventional treatments (e.g., heat therapy, physical modalities, exercise, or stretching) were included only if these co-interventions were applied equally to both groups. Studies were excluded if they involved high-velocity, low-amplitude thrust manipulation; incorporated soft tissue mobilization; or assigned MET and JM in combination within a single intervention arm.
• Outcome measures: The primary outcome was pain intensity, measured using the Visual Analogue Scale (VAS) or Numeric Pain Rating Scale (NPRS). Secondary outcomes included neck-related disability (e.g., Neck Disability Index [NDI]) and cervical range of motion (ROM) measured using a goniometer or inclinometer. Outcome measures were required to be assessed at baseline and post-intervention. Studies reporting outcomes only at follow-up without baseline or immediate post-treatment assessment were excluded.
• Study designs: Only randomized controlled trials (RCTs) were included. Non-randomized or non-comparative studies were excluded.

### 2.2. Literature Searches and Screening Process

A systematic literature search was conducted from inception up to September 12, 2025, across PubMed, the Cochrane Library, and EMBASE, without publication year restrictions. Citation tracking was performed by screening the reference lists of retrieved records to identify additional relevant literature. Efforts were made to obtain full texts for all eligible works, including theses, dissertations, and unpublished studies. All identified records were screened independently by two reviewers (L-JW and D-WK). Search terms included combinations of neck pain conditions (MNP, nonspecific neck pain, cervical pain), MET and its variants (PIR and RI), and joint mobilization techniques (Maitland, Cyriax, Mulligan SNAGs and NAGs). The search was conducted using Medical Subject Headings (MeSH) in PubMed and equivalent terms in other databases. Owing to the absence of specific MeSH descriptors for MET and JM, we used the broader headings to encompass these specific techniques. Full search strategies for each database are reported in Supplementary Table S1.

Manual reference screening of eligible full-text articles was additionally performed to capture studies not detected by electronic searching. Duplicate records were identified and removed using Zotero (version 7.0.32; Corporation for Digital Scholarship, Vienna, VA, USA). Any disagreements were resolved through discussion, and if necessary, arbitration was provided by a third reviewer (E-JL). The overall screening and study selection procedures were documented in a PRISMA flowchart consistent with the PRISMA 2020 reporting standards [19].

### 2.3. Data Extraction

Two reviewers (L-JW and D-WK) independently extracted data using a pre-piloted standardized form. Any discrepancies between the reviewers were addressed through discussion, and unresolved cases were adjudicated by a third reviewer (E-JL).

Extracted information included study-level data (authors, publication year, country), participant characteristics (sample size, age, sex distribution, and symptom duration), intervention protocol details (MET and JM procedures, duration, and treatment schedule), and reported clinical outcomes (pain ratings, disability measures, and cervical ROM). Post-intervention summary statistics, including means and standard deviations, were collected for quantitative synthesis. Missing data were not imputed.

### 2.4. Risk of Bias Assessment

Risk of bias was assessed using the revised Cochrane Risk of Bias tool (RoB 2.0), following guidance from the Cochrane Handbook for Systematic Reviews of Interventions [20,21]. Two reviewers (L-JW and D-WK) independently evaluated five domains: the randomization process, deviations from intended interventions, missing outcome data, outcome measurement, and selective reporting. Each domain was rated as “low risk,” “some concerns,” or “high risk,” and an overall risk-of-bias judgment was determined using the RoB 2.0 algorithm (Risk-of-Bias VISualization; developed by Luke McGuinness; University of Bristol, Bristol, UK) [22]. Disagreements were resolved through discussion, with consultation of a third reviewer (E-JL) when necessary. When information in the original trial reports was insufficient to determine whether deviations from intended interventions or missing outcome data could have introduced bias, the corresponding domains were judged as having “some concerns,” rather than assuming that bias had occurred.

### 2.5. Data Synthesis and Analysis

Quantitative synthesis was conducted using Review Manager (RevMan) Version 5.4 (Cochrane Collaboration, Copenhagen, Denmark). A random-effects model was prespecified to account for anticipated clinical and methodological heterogeneity. Pooled effects were presented as forest plots with 95% confidence intervals (CIs), and statistical significance was set at *p* < 0.05. Between-study heterogeneity was assessed using the *I*^2^ statistic and interpreted as low (≤25%), moderate (26–50%), substantial (51–75%), or considerable (>75%) [23].

For continuous outcomes, mean difference (MD) was used when measurement scales were consistent, and standardized mean difference (SMD) when they differed. Pain scores were standardized to a 0–10 scale; both the mean and SD of VAS scores reported in millimeters were divided by 10 to align with NPRS scores. Disability outcomes were analyzed using SMD owing to varying instruments, whereas cervical ROM, measured in degrees, was analyzed using MD.

Effect estimates were calculated as MET minus JM. For cervical ROM, group means were multiplied by −1 before analysis, with SDs unchanged, to align effect direction with pain and disability. Thus, positive estimates favored JM and negative estimates favored MET across all outcomes.

Baseline pain severity was categorized as mild (≤3.4), moderate (3.5–6.4), or severe (≥6.5) [24]. Published minimal clinically important difference (MCID) thresholds of 2 points for the NPRS [25] and 5 points for the NDI (0–50 scale, equivalent to 10% points on a 0–100% scale) [26] were used to contextualize within-group changes in the respective instruments, not to determine between-group superiority. These thresholds were not applied to pooled between-group effects or used in the GRADE assessment of imprecision.

No validated MCID applicable to this population was identified for the CNFDS [27], and no instrument-specific MCID was applied to the pooled disability SMD. An approximate 10° threshold for cervical flexion and extension was used descriptively for within-group changes, rather than as a validated MCID [28].

### 2.6. Subgroup Analysis

• Subgroup analyses were conducted to explore potential sources of clinical heterogeneity. These analyses were conducted post hoc for exploratory purposes. Formal tests for subgroup differences were performed without adjustment for multiple comparisons.
• Mobilization technique type: This grouping broadly distinguished conventional passive mobilization approaches from Mulligan-based protocols emphasizing a pain-free principle and active movement to explore potential sources of heterogeneity. However, Maitland and Cyriax are distinct techniques, and protocols within each category varied.
• Intervention duration in Mulligan trials: Two-week versus four-week treatment protocols. This analysis was intended to explore whether treatment duration might contribute to differences in treatment effects, as previous manual therapy research has suggested that treatment duration may influence the magnitude of clinical improvement [29].

### 2.7. Sensitivity Analysis

Sensitivity analyses were conducted using a leave-one-out approach to evaluate the consistency of the meta-analysis results. Each study was iteratively excluded to assess its individual influence on the overall effect estimates and to identify potential sources of statistical heterogeneity. These analyses assessed the influence of individual studies on the pooled estimates but could not establish that the sources of heterogeneity had been identified or resolved.

### 2.8. GRADE Assessment

The certainty of evidence for each outcome was evaluated using the Grading of Recommendations, Assessment, Development, and Evaluation (GRADE) methodology via GRADE pro GDT (McMaster University, developed by Evidence Prime, Hamilton, ON, Canada) [30]. The evidence, initially set to high for randomized controlled trials, was downgraded across five domains: risk of bias, inconsistency, indirectness, imprecision, and publication bias. The evidence was ultimately categorized into four levels: high, moderate, low, or very low. Subgroup-specific GRADE assessments were also conducted for protocols demonstrating lower heterogeneity. Two reviewers (L-JW and D-WK) independently performed these assessments, with disagreements resolved through consultation with a third reviewer (E-JL). Imprecision was evaluated primarily using the confidence interval (CI) approach. Evidence was downgraded by one level if the 95% CI crossed the line of no effect. According to recent GRADE guidance, additional downgrading based on the optimal information size (OIS) was not applied to the primary analyses, as the total sample sizes (*n* = 250) exceeded the minimal required threshold (30% of OIS, *n* = 240) for continuous outcomes.

For the GRADE risk-of-bias domain, RoB 2 judgments were considered in relation to the potential impact of bias on the pooled comparative effect estimates. A judgment of “some concerns” in RoB 2 did not automatically warrant downgrading. Because both MET and JM were active manual therapies, nonspecific treatment expectations could have affected both groups, potentially reducing differential bias compared with active-treatment versus no-treatment comparisons. Nevertheless, differences in treatment expectations could not be excluded. The potential influence of outcome measurement, deviations from intended interventions, and missing outcome data was considered when determining whether risk of bias warranted downgrading.

## 3. Results

### 3.1. Study Selection

**Figure 1:**
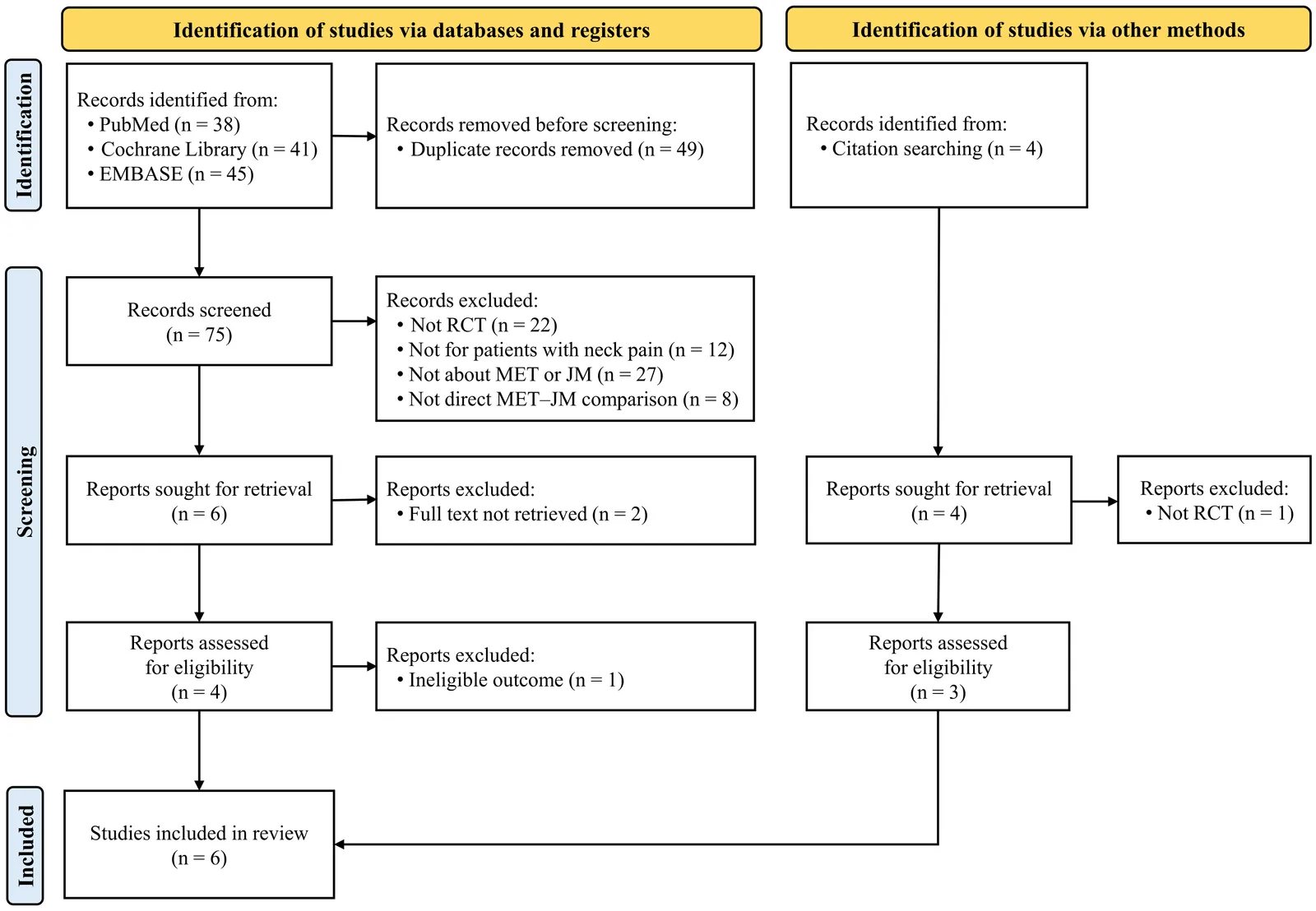
Flow diagram of the study selection process. MET, muscle energy technique; JM, joint mobilization.

Overall, 124 records were retrieved from PubMed, the Cochrane Library, and EMBASE. After duplicate removal, 75 records underwent title and abstract screening. A total of seven studies were assessed through full-text review, of which one study was excluded because of ineligible outcome measures (Supplementary Table S2). Ultimately, six RCTs satisfied all predefined eligibility criteria and were included in the review. The study selection process is illustrated using a PRISMA 2020-compliant flow diagram (Figure 1), generated with the PRISMA 2020 tool (Shiny web application; Haddaway et al., 2022) [31].

### 3.2. Study Characteristics

The six included trials encompassed 381 participants: 209 women, 132 men, and 40 with unreported sex information [32]. Sex distribution across treatment arms was comparable within each study. The sample size-weighted (N-weighted) mean age of enrolled participants was 40.16 ± 12.30 years, although two RCTs did not present age-related baseline characteristics [32,33]. The N-weighted mean symptom duration was 16.09 ± 22.73 months. Most participants reported symptoms persisting for at least 3 months, though three studies [32,33,34] did not clarify duration thresholds.

Of the 381 participants, 80 were assigned to additional conventional-treatment-only groups and were therefore excluded from the direct MET–JM comparisons. The remaining 301 participants contributed to the pain and disability analyses, while 250 contributed to the cervical ROM analyses because one trial did not report ROM outcomes [35]. One trial recruited patients with clinically and radiographically supported cervical spondylosis and neck pain lasting >3 months, excluding those with arm radiation or neurological disease [35]. Baseline pain intensity ranged from 5.12 to 8.90 on a standardized 0–10 scale, with an N-weighted mean of 6.83 ± 1.82, indicating a moderate-to-severe symptom level. Baseline disability scores ranged from 12.92 to 47.83 across different instruments. Excluding one study [35] that did not report ROM values, baseline cervical flexion ROM ranged from 34.47° to 62.25° (N-weighted mean: 42.19 ± 10.90°), whereas extension ROM ranged from 21.14° to 54.50° (N-weighted mean: 40.66 ± 10.69°). The participant characteristics and outcome data of each included study are presented in Table 1, with detailed extracted values provided in Supplementary Table S3.

#### 3.2.1. Interventions and Comparators

All included studies compared MET with JM. MET interventions consistently employed the PIR technique; however, contraction intensity, treatment dosage, and intervention duration varied among studies. MET protocols generally incorporated low-intensity isometric contractions at approximately 20–50% of maximal voluntary contraction, with contraction hold times ranging from 5 to 10 s and 3–5 repetitions per session. Four studies specifically targeted the upper trapezius, sternocleidomastoid, scalene, and levator scapulae muscles, whereas two studies did not report the targeted musculature [32,37]. Treatment sessions were delivered three to four times per week over a period of 2–4 weeks, resulting in a total of 6–16 sessions (Supplementary Table S3).

JM protocols varied across studies and included Cyriax mobilization [35], Maitland mobilization [36], Mulligan techniques [32,33,37], and a combined Maitland–Mulligan approach [34]. In the combined approach, Maitland mobilizations were applied to painful segments, whereas Mulligan SNAGs were used to address hypomobile segments. The intervention period was either 4 weeks (*n* = 4) or 2 weeks (*n* = 2).

#### 3.2.2. Outcome Measures

Pain intensity was assessed using VAS in four studies [32,34,35,36], and NPRS in two studies [33,37]. Disability was assessed using the NDI in five trials [32,33,34,36,37], and the Copenhagen Neck Functional Disability Scale (CNFDS) in one [35]. Five studies evaluated cervical ROM using goniometric or inclinometric methods [32,33,34,36,37].

#### 3.2.3. Study Design and Geographic Distribution

All included trials used a parallel-group RCT design. Three studies were conducted in Pakistan [33,34,37], with one study each from Türkiye [35], Egypt [36], and India [32]. A detailed summary of participant demographics, intervention characteristics, and outcome measurement tools is presented in Table 1. Outcomes were assessed at baseline and immediately after treatment, following completion of the 2- or 4-week intervention program. No follow-up data were available.

### 3.3. Primary Outcome: Pain

**Figure 2:**
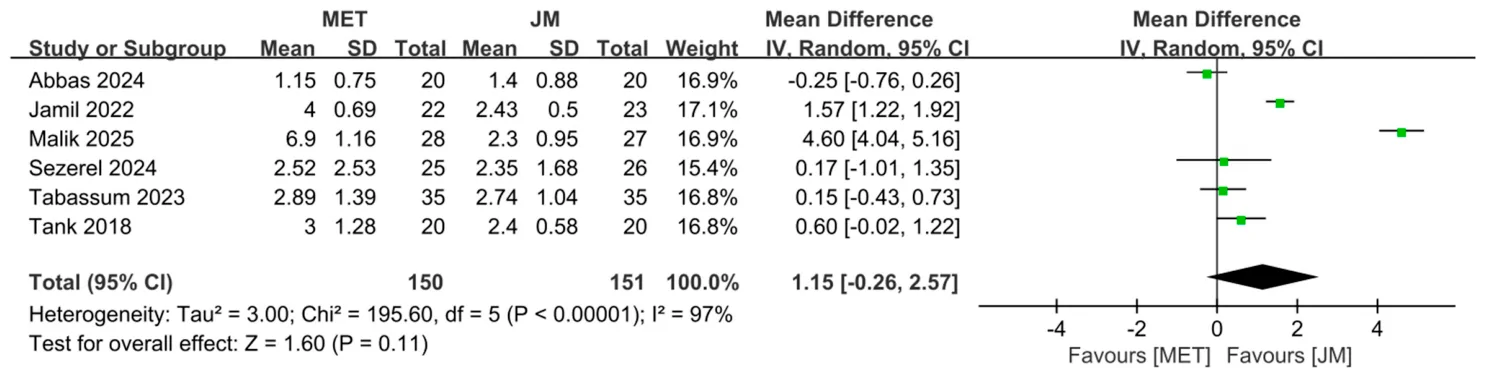
Comparison of pain scores between MET and JM [32,33,34,35,36,37]. Pain scores from VAS and NPRS were harmonized to a 0–10 scale (VAS means and SDs divided by 10 where reported in mm) and pooled using mean differences (MDs). MET, muscle energy technique; JM, joint mobilization; VAS, Visual Analogue Scale; NPRS, Numeric Pain Rating Scale.

**Figure 3:**
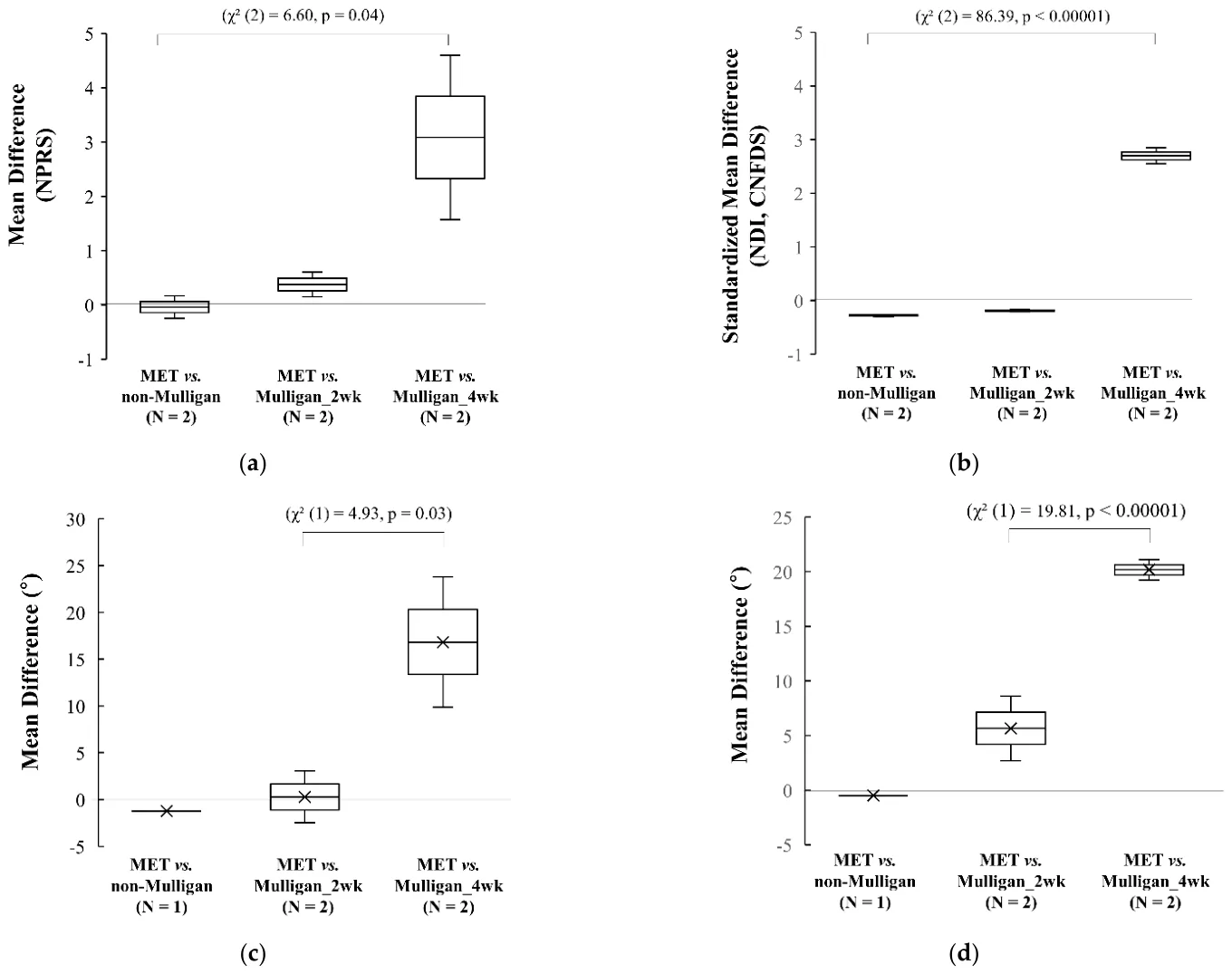
Comparison between MET and subgroups of JM in pain, disability, and ROM. (**a**) Pain. (**b**) Disability. (**c**) Flexion ROM. (**d**) Extension ROM. Numbers in parentheses refer to the number of included studies. Positive values indicate greater improvement with JM, while negative values indicate greater improvement with MET. MET, muscle energy technique; JM, joint mobilization; MD, mean difference; SMD, standardized mean difference; NDI, Neck Disability Index; CNFDS, Copenhagen Neck Functional Disability Scale; ROM, range of motion.

The pooled analysis did not identify a statistically significant between-group difference in pain (MD = 1.15; 95% CI −0.26 to 2.57; *p* = 0.11), with considerable heterogeneity (*I*^2^ = 97%) (Figure 2). Most individual treatment groups demonstrated within-group improvements exceeding the published MCID threshold for pain, with the exception of the MET group in one study [37] (Supplementary Table S3). However, these within-group improvements do not establish comparative superiority between MET and JM. Sensitivity analyses did not substantially alter heterogeneity or the direction of the pooled effects (Supplementary Table S5). Subgroup analyses showed that 4-week Mulligan interventions yielded more favorable pain reduction than MET (MD = 3.08; 95% CI 0.11 to 6.05; *p* = 0.04), though heterogeneity remained high (*I*^2^ = 99%) (Figure 3 and Supplementary Table S9).

### 3.4. Secondary Outcomes

#### 3.4.1. Disability

**Figure 4:**
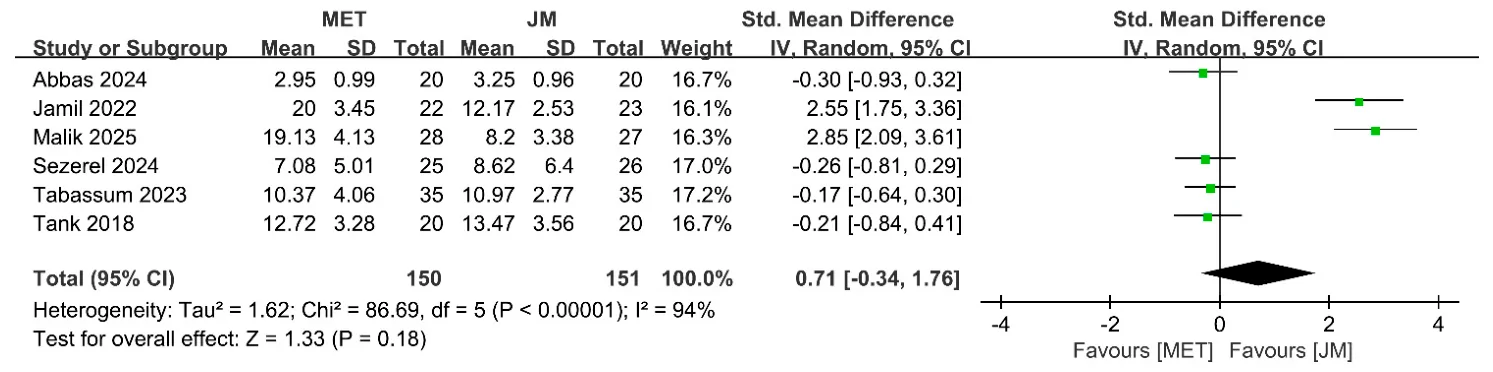
Comparison of disability scores between MET and JM (NDI, CNFDS) [32,33,34,35,36,37]. MET, muscle energy technique; JM, joint mobilization; NDI, Neck Disability Index; CNFDS, Copenhagen Neck Functional Disability Scale.

The pooled analysis did not identify a statistically significant between-group difference in disability, with considerable heterogeneity (Figure 4). Improvements in NDI scores within individual treatment groups generally exceeded the MCID threshold; however, between-group differences between JM and MET remained inconsistent. Clinical relevance could not be established for the CNFDS-based outcome, as no validated MCID was available. Sensitivity analyses did not meaningfully alter pooled outcomes or heterogeneity patterns (Supplementary Table S6). In the exploratory subgroup analysis of two trials, the pooled estimate favored JM for disability improvement (SMD = 2.71; 95% CI 2.15 to 3.26; *I*^2^ = 0%). However, this finding should be interpreted cautiously because of the small number of trials and potential confounding by study characteristics (Figure 3 and Supplementary Table S9).

#### 3.4.2. Cervical ROM

**Figure 5:**
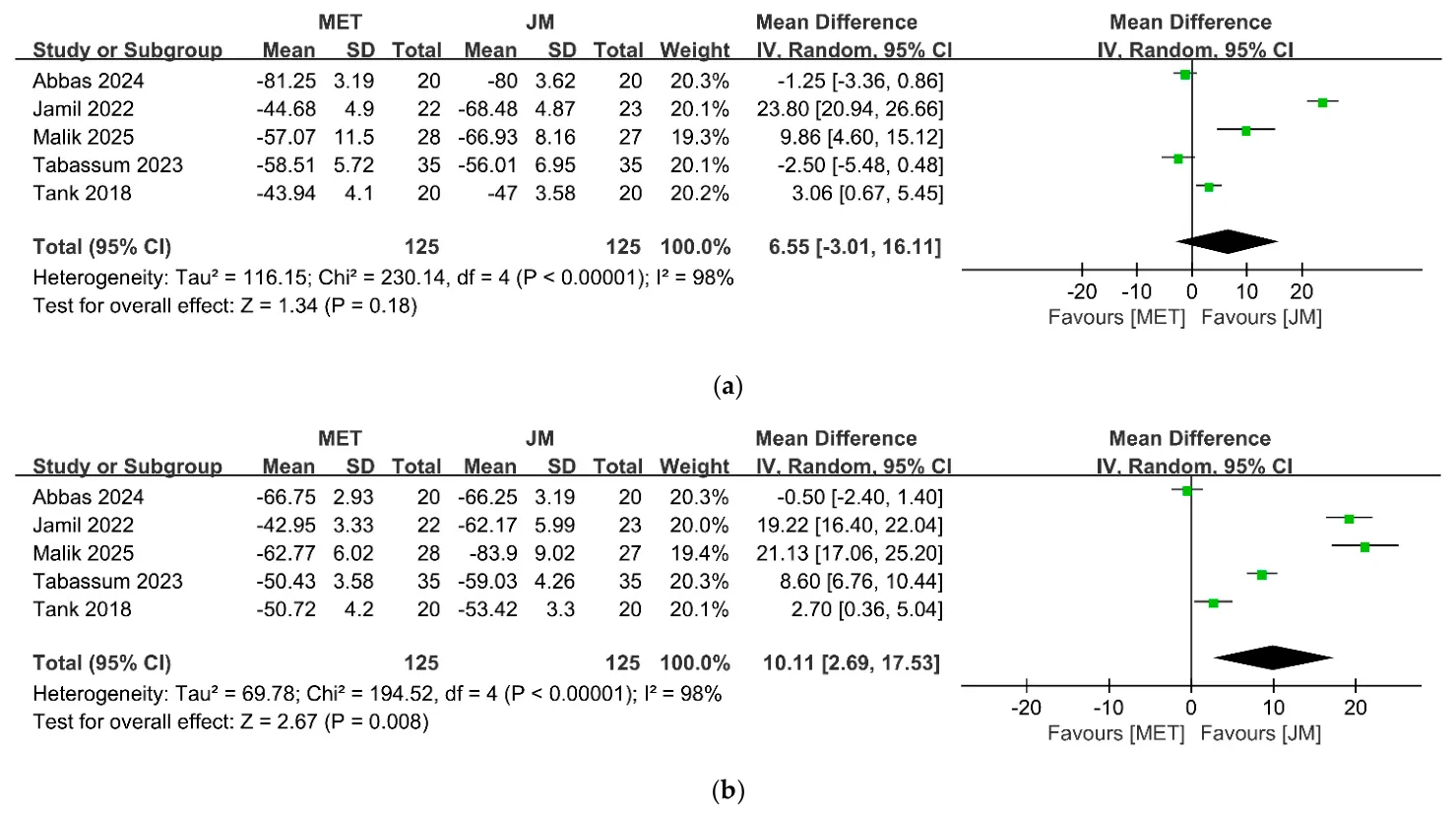
Comparison of ROM between MET and JM (°). (**a**) Flexion ROM. (**b**) Extension ROM [32,33,34,35,36,37]. MET, Muscle Energy Technique; JM, joint mobilization; ROM, range of motion.

The pooled analysis did not identify a statistically significant between-group difference in flexion ROM, with considerable heterogeneity (Figure 5). Most intervention groups demonstrated within-group improvements that exceeded the predefined interpretive threshold for flexion ROM (10°); however, this threshold was not reached by the MET group in one study [33], or by either the MET or JM groups in another study (Supplementary Table S3) [32]. Sensitivity analyses supported the robustness of pooled estimates (Supplementary Table S7). Subgroup analyses suggested that 4-week Mulligan mobilization protocols were associated with greater gains in flexion ROM compared with MET, although heterogeneity remained substantial (Figure 3 and Supplementary Table S9).

Extension ROM favored JM over MET, again with notable heterogeneity (Figure 5). All included study groups demonstrated improvements exceeding the prespecified interpretive threshold for extension ROM (10°). Sensitivity analyses indicated that the direction of the pooled effect remained consistent (Supplementary Table S8). Subgroup analyses demonstrated that 4-week Mulligan interventions were associated with further improvements, with negligible heterogeneity (*I*^2^ = 0%) (Figure 3 and Supplementary Table S9).

### 3.5. Quality Assessment

**Figure 6:**
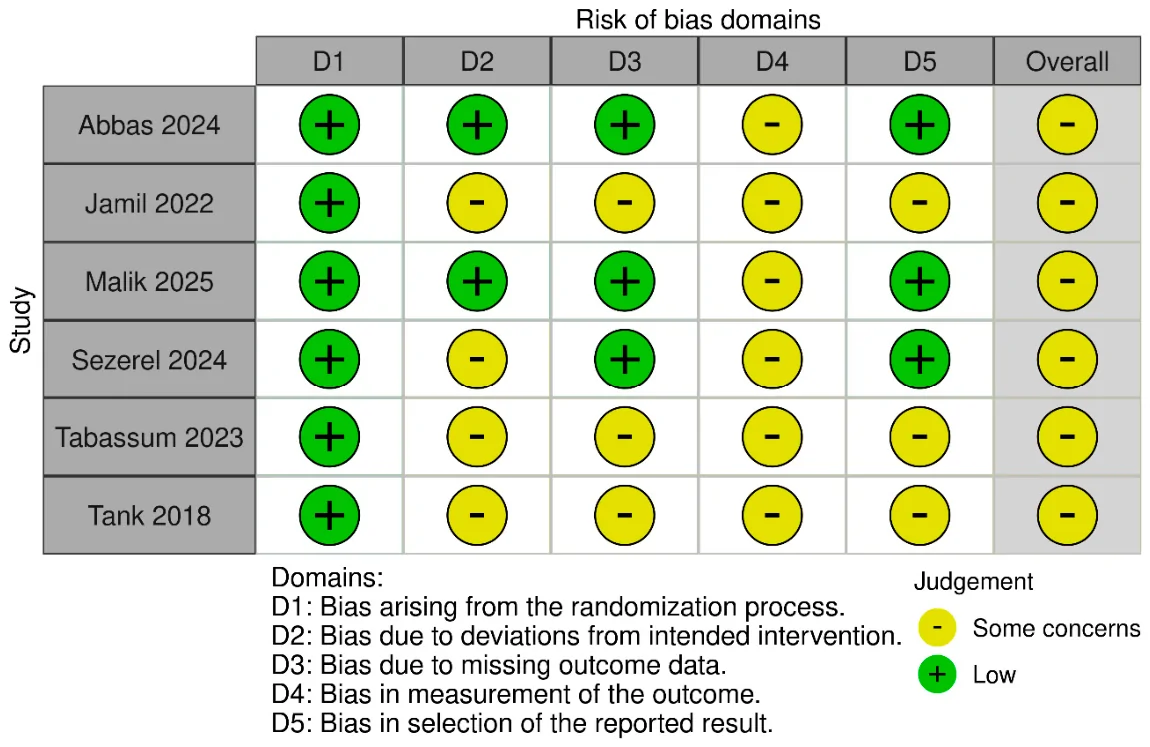
Summary of risk of bias assessment [32,33,34,35,36,37].

All six studies were judged as having “some concerns” overall in the RoB 2 assessment (Figure 6) [32,33,34,35,36,37], primarily due to Domain 4 (bias in measurement of the outcome). For patient-reported pain and disability, participants themselves were the outcome assessors. However, because both MET and JM were active manual therapies, between-group differences in treatment expectations and nonspecific effects may have been smaller than in comparisons with no-treatment controls. Nevertheless, potential measurement bias could not be excluded because the interventions were distinguishable and treatment expectations were not assessed. Therefore, Domain 4 was rated as having “some concerns.” For cervical ROM, absent or insufficiently reported assessor blinding also raised concerns about measurement bias.

In Domain 2 (bias due to deviations from intended interventions), four studies were rated as having “some concerns” [32,33,34,35]. Although lack of participant blinding does not necessarily indicate intervention deviations, insufficient reporting prevented determination of whether such deviations occurred or whether appropriate analytical methods, including intention-to-treat analysis, were used. Thus, these judgments reflected uncertainty due to insufficient reporting rather than confirmed deviations or bias.

The certainty of evidence for the overall analyses comparing JM and MET ranged from “very low” to “moderate” (Table 2). Pain, disability, and flexion ROM were rated as “very low,” downgraded for very serious inconsistency and serious imprecision. By contrast, extension ROM was rated as “moderate”. Although heterogeneity was considerable, effect directions remained consistent, and confidence intervals overlapped, resulting in a downgrade of only one level for inconsistency. No serious concerns were identified regarding indirectness. Publication bias could not be reliably assessed because of the small number of studies and therefore cannot be excluded. In the 4-week Mulligan subgroup analyses, the certainty of evidence varied across outcomes. Pain was rated as “very low” owing to very serious inconsistency. Despite the limited number of studies (*n* = 2) and total sample size (*n* = 100), disability and extension ROM were rated as “moderate” because the substantial effect size and consistent direction of improvement favored the 4-week Mulligan group without further reasons for downgrading. Flexion ROM was rated as “low” after downgrading for serious inconsistency and serious imprecision.

## 4. Discussion

### 4.1. Summary of Main Findings

This systematic review included six RCTs directly comparing MET and JM for mechanical neck pain. Although within-group improvements were observed in nearly all treatment arms, the pooled between-group differences in pain and disability were small and not statistically significant. No statistically significant difference was identified in cervical flexion ROM, whereas cervical extension ROM favored JM. Improvement from baseline does not establish the superiority of either technique, and non-significant between-group differences should not be interpreted as evidence of equivalence. Considerable heterogeneity further limits the interpretability and generalizability of these pooled estimates.

Subgroup analyses suggested effects favoring 4-week Mulligan mobilization for some outcomes. However, heterogeneity remained considerable for pain (*I*^2^ = 99%), indicating that stratification by technique and duration did not adequately explain the variability in this outcome. Although disability and extension ROM showed no observed statistical heterogeneity within this subgroup (*I*^2^ = 0%), these estimates were based on only two trials and do not establish that treatment duration or mobilization technique explains the differences across studies. These findings should therefore be regarded as exploratory.

### 4.2. Comparison with Previous Research

Sbardella et al. reported benefits of MET for pain in acute neck pain and cervical ROM in chronic neck pain, particularly when combined with conventional rehabilitation [38]. Hidalgo et al. reported benefits of manual therapy combined with exercise for pain and function in nonspecific neck pain [39]. Consistent with previous literature, within-group improvements were observed following both MET and JM. However, these improvements do not establish the comparative superiority of either intervention. Prior systematic reviews have generally evaluated MET and JM separately or compared them with conventional physiotherapy [38,39], whereas direct comparisons between the two techniques have been limited.

Although subgroup analyses identified pronounced improvements in trials employing a 4-week Mulligan protocol, these findings must be interpreted with caution. Given the limited number of studies, it remains difficult to separate whether the observed benefits stem from longer treatment duration, characteristics of the Mulligan technique itself, or other trial factors. Nevertheless, previous studies indicate that manual therapies involving active movement—such as Mulligan mobilization—enhance sensorimotor integration and neurophysiological pain control, contributing to kinematic and functional recovery [40]. Although previous literature has proposed sensorimotor and neurophysiological mechanisms underlying manual therapy, these mechanisms were not directly assessed in the included trials. Therefore, the observed subgroup differences cannot be attributed to treatment duration or specific physiological mechanisms. However, this remains hypothesis-generating and warrants verification in future trials designed to isolate duration from technique-specific effects.

### 4.3. Clinical and Scientific Implications

The pattern observed in the 4-week Mulligan subgroup raises the possibility that this technique may influence sensorimotor function differently from MET when applied repeatedly over time. Patients with MNP often present with disrupted proprioception, which may contribute to sensorimotor incongruence and persistent muscle overactivity [41]. These disturbances contribute to ongoing pain and restricted cervical motion [42].

Mulligan mobilization applies an accessory glide concurrently with pain-free active movement [14]. While Hing et al. outlined the clinical tenets of Mulligan mobilization [43], broader neurophysiological studies, such as Ostry et al. [44], show that active motor learning can modulate sensory functions. Whether these principles translate into mechanoreceptor stimulation during manual therapies remains a plausible but untested hypothesis. In addition, the active movement component may engage motor and somatosensory cortical areas, potentially contributing to improved body position sense [45].

A crucial distinction between the two techniques is that Mulligan mobilization is generally performed within a pain-free range. Vlaeyen et al. and Moseley et al. provide a theoretical rationale for pain-free movement to reduce movement-related fear [46,47]. Repeated pain-free movement has also been proposed to facilitate motor learning and neurophysiological adaptation, processes that likely require reinforcement over time [48].

In the present review, differences between JM and MET were minimal following shorter interventions, whereas some favorable findings were observed in studies employing a 4-week Mulligan protocol. This pattern may reflect the possibility that the repeated, guided, pain-free movements characteristic of Mulligan mobilization facilitate gradual motor relearning and soft-tissue adaptation over time [49]. Previous comparative studies have similarly reported negligible differences following short-term interventions (<4 weeks) [49,50], whereas some studies with longer treatment durations (≥4 weeks) have suggested more favorable outcomes with Mulligan mobilization [51,52]. However, these mechanisms were not directly evaluated in the included studies, and the observed differences may also reflect study-specific factors. Potential sources include differences in diagnostic criteria and clinical presentations, symptom duration, baseline symptom severity, treatment parameters, therapist expertise, and the types of co-interventions used across studies. Therefore, these interpretations should be considered hypothesis-generating and require further investigation.

Taken together, the exploratory subgroup findings suggest possible differences in treatment effects across intervention protocols. However, the limited number of trials, substantial heterogeneity, and potential confounding by trial characteristics preclude conclusions regarding the superiority of Mulligan mobilization or the optimal treatment duration. MET may likewise remain a useful option for symptom management, particularly when immediate intervention is prioritized.

### 4.4. Strengths and Limitations

This review has two notable strengths. To our knowledge, it is the first systematic review to directly compare MET and JM for neck pain within a unified analytic framework. In addition, the exploratory duration-related pattern in the 4-week Mulligan subgroup may inform future research, although it requires confirmation.

Several limitations should be acknowledged. First, only six RCTs with a small total sample were included; the certainty of the evidence was therefore downgraded by GRADE, and firm clinical recommendations cannot be made. Second, heterogeneity was considerable for most outcomes. Potential sources include differences in diagnostic criteria, symptom duration, baseline severity, treatment parameters (e.g., target muscles, contraction intensity, hold duration, and repetitions), and co-interventions, which were balanced between groups within each trial but differed across trials. Subgroup and leave-one-out analyses did not adequately explain this variability, particularly for pain in the 4-week Mulligan subgroup (*I*^2^ = 99%), and meta-regression was not feasible because of the small number of trials and incomplete reporting. The pooled estimates therefore represent average effects across heterogeneous trials, not definitive evidence of comparative superiority or equivalence. In addition, although NDI and CNFDS were pooled using SMD to assess neck-related functional disability, differences in their item content and measurement properties may limit the comparability of these instruments and the interpretation of the pooled disability estimate.

Third, all studies had some concerns regarding risk of bias, particularly in outcome measurement. Because pain and disability were self-reported, treatment expectations may have influenced the results, including the findings favoring 4-week Mulligan mobilization, although the direction and magnitude of this influence are unknown. Fourth, cervical mobility was assessed only in the sagittal plane (flexion and extension) because lateral flexion and axial rotation were insufficiently reported, precluding evaluation of multi-planar kinematics. Fifth, all studies were conducted in the Middle East or South Asia, which limits generalizability. Finally, publication bias cannot be excluded. Each analysis included only five or six studies, which limited the usefulness of funnel plot asymmetry tests, and unpublished unfavorable or non-significant studies could lead to overestimation of treatment benefits. Furthermore, the subgroup analyses were conducted post hoc, with only one or two studies per subgroup. No adjustment for multiple comparisons was applied, increasing the risk of false-positive findings. The clinical heterogeneity within subgroups and potential confounding between mobilization technique and treatment duration further limit interpretation. Therefore, these findings should be considered exploratory and hypothesis-generating rather than confirmatory.

### 4.5. Implications for Future Research

Future trials should adopt standardized intervention protocols with explicitly defined treatment parameters and target regions, incorporate adequate blinding and allocation concealment, and include larger sample sizes to reduce imprecision. Direct measurements of sensory–motor or proprioceptive function may help clarify the mechanisms underlying treatment differences. Furthermore, factorial designs could disentangle technique and duration effects, while longer-term follow-up could assess the persistence of treatment effects.

## 5. Conclusions

The available evidence does not establish the superiority of either MET or JM for improving pain, disability, or cervical ROM in patients with MNP, and a non-significant difference should not be interpreted as equivalence. Exploratory analyses suggested larger point estimates favoring JM in two 4-week Mulligan trials, but these rested on few studies, were confounded by trial characteristics, and were affected by substantial heterogeneity. The certainty of evidence was very low for most outcomes. Adequately powered randomized trials with standardized protocols, clearly defined populations, blinded outcome assessment, and follow-up are warranted.

## Figures and Tables

**Table 1:** Characteristics and key findings of included studies.

Author, Year (Country)	Design	Intervention	N. of Participants (F/M)	Age (Years)	Symptom Length (Month)	Treatment Duration	Outcome Measure	Result
Abbas, 2024 (Egypt) [36]	3-arm	MET + CT JM (Maitland) + CT CT	20 (12/8) 20 (13/7) 20 (11/9)	24.55 ± 7.73 23.15 ± 5.74 24.30 ± 7.69	9.25 ± 3.07 8.90 ± 3.16 9.25 ± 3.38	3 sessions/wk for 4 weeks	(1) Pain: VAS (2) Disability: NDI (3) ROM: all directions	• Pain/NDI/ROM : MET, JM > CT **
Jamil, 2022 (Pakistan) [33]	2-arm	MET + CT JM (NAGs) + CT	22 (13/9) 23 (7/16)	NR	NR	4 sessions/wk for 4 weeks	(1) Pain: NPRS (2) Disability: NDI (3) ROM: Flex, Ext	• Pain/NDI/ROM : JM > MET **
Malik, 2025 (Pakistan) [37]	2-arm	MET + CT JM (SNAGs) + CT	27 (15/12) 28 (16/12)	40.90 ± 11.10 41.70 ± 10.50	8.10 ± 3.60 8.30 ± 3.70	3 sessions/wk for 4 weeks	(1) Pain: NPRS (2) Disability: NDI (3) ROM: Flex, Ext	• Pain/NDI/ROM : JM > MET **
Sezerel, 2024 (Türkiye) [35]	3-arm	MET + ET JM (Cyriax) + ET ET	25 (17/8) 26 (23/3) 25 (15/10)	51.60 ± 7.64 50.54 ± 8.43 55.48 ± 8.24	21.16 ± 25.73 32.69 ± 39.41 27.80 ± 32.02	3 sessions/wk for 4 weeks	(1) Pain: VAS (2) Disability: CNFDS	• Pain/CNFDS: ns
Tabassum, 2023 (Pakistan) [34]	3-arm	MET + CT JM + CT (Maitland; SNAGs) CT	35 (NR) 35 (NR) 35 (NR)	40.09 ± 4.29 40.14 ± 4.57 39.26 ± 5.19	≥3	3 sessions/wk for 2 weeks	(1) Pain: VAS (2) Disability: NDI (3) ROM: all directions	• Pain/NDI: MET, JM > CT * • Flex, Rot, Side bending : MET > JM * • Ext: JM > MET *
Tank, 2018 (India) [32]	2-arm	MET + CT JM (SNAGs) + CT	20 (NR) 20 (NR)	18–45	<3	3 sessions/wk for 2 weeks	(1) Pain: VAS (2) Disability: NDI (3) ROM: all directions	• Pain/NDI/ROM : ns
Summary	6RCTs (2-arm:3, 3-arm:3)		381 (F/M/NR) 209/132/40	40.16 ± 12.30	16.09 ± 22.73	10.2 ± 3.5 sessions for 3.2 ± 1.0 weeks		

MET, Muscle Energy Technique; JM, Joint Mobilization; CT, Conventional Therapy; F, female; M, male; wk, week (s); VAS, Visual Analogue Scale; NDI, Neck Disability Index; ROM, cervical range of motion; NAGs, Natural Apophyseal Glides; NR, Not Reported; Flex, flexion; Ext, extension; SNAGs, Sustained Natural Apophyseal Glides; NPRS, Numeric Pain Rating Scale; ET, Electro-Thermal Therapy; CNFDS, Copenhagen Neck Functional Disability Scale; ns, no significant difference between the two groups; Rot, rotation. Only inter-group results are presented in this table. Intra-group analyses in all studies demonstrated statistically significant improvements from pre- to post-treatment (*p* < 0.05). *, *p* < 0.05; **, *p* < 0.01.

**Table 2:** Summary of certainty of evidence.

Effect of MET vs. JM on	N. of Participants (N. of RCTs)	Effect Size (95% CI)	Risk of Bias	Inconsistency	Indirectness	Imprecision	Other Considerations	Quality of Evidence
Pain	301 (6)	MD = 1.15 (−0.26 to 2.57)	not serious	very serious ^a^	not serious	serious ^c^	none	㊉㊀㊀㊀ Very low
Disability	301 (6)	SMD = 0.71 (−0.34 to 1.76)	not serious	very serious ^a^	not serious	serious ^c^	none	㊉㊀㊀㊀ Very low
Flexion ROM	250 (5)	MD = 6.55 (−3.01 to 16.11)	not serious	very serious ^a^	not serious	serious ^c^	none	㊉㊀㊀㊀ Very low
Extension ROM	250 (5)	MD = 10.11 (2.69 to 17.53)	not serious	serious ^b^	not serious	not serious	none	㊉㊉㊉㊀ Moderate
Pain (MET vs. 4 wks Mulligan)	100 (2)	MD = 3.08 (0.11 to 6.05)	not serious	very serious ^a^	not serious	serious ^d^	none	㊉㊀㊀㊀ Very low
Disability (MET vs. 4 wks Mulligan)	100 (2)	SMD = 2.71 (2.15 to 3.26)	not serious	not serious	not serious	serious ^d^	none	㊉㊉㊉㊀ Moderate
Flexion ROM (MET vs. 4 wks Mulligan)	100 (2)	MD = 17.01 (3.36 to 30.67)	not serious	serious ^b^	not serious	serious ^d^	none	㊉㊉㊀㊀ Low
Extension ROM (MET vs. 4 wks Mulligan)	100 (2)	MD = 19.84 (17.52 to 22.15)	not serious	not serious	not serious	serious ^d^	none	㊉㊉㊉㊀ Moderate

CI, confidence interval; MD, mean difference; SMD, standardized mean difference; MCID, minimal clinically important difference. ^a^, Downgraded by two levels for inconsistency; Effect directions were opposite, confidence intervals did not overlap, and heterogeneity remained unexplained by subgroup or sensitivity analyses; ^b^, Downgraded by one level for inconsistency; Although heterogeneity was considerable (I^2^ > 90%), effect directions were consistent and CIs overlapped; ^c^, Downgraded by one level for imprecision because the 95% confidence interval crossed the line of no effect and encompassed a range of potentially clinically relevant effects, indicating uncertainty regarding the magnitude and direction of the true between-group difference; ^d^, Downgraded by one level for imprecision because the subgroup analysis included only 100 participants from two trials, resulting in an insufficient information size to support a precise effect estimate. The symbols indicate the certainty of evidence according to the GRADE approach: ⊕⊕⊕⊖, moderate; ⊕⊕⊖⊖, low; and ⊕⊖⊖⊖, very low.

## Data Availability

The data that support the findings of this study are available from the corresponding author upon reasonable request.

## Supplementary Materials

The following supporting information can be downloaded at: https://www.mdpi.com/article/10.3390/healthcare14213719/s1, Table S1: Search strategies; Table S2: Excluded studies after full-text review; Table S3: Data Extraction for Meta-Analysis; Table S4: Intervention protocols of included studies; Table S5: Sensitivity analysis of Pain intensity (leave-one-out method); Table S6: Sensitivity analysis of Disability (leave-one-out method); Table S7: Sensitivity analysis of flexion ROM (leave-one-out method); Table S8: Sensitivity analysis of extension ROM (leave-one-out method); Table S9: Comparison of Pooled Estimates and Heterogeneity: Overall Analysis vs. 4-week Mulligan Subgroup; Figure S1: MET versus Subgroups of JM (non-Mulligan, Mulligan) in pain; Figure S2: MET versus Subgroups of JM(non-Mulligan, Mulligan) in disability; Figure S3: MET versus Subgroups of JM(Mulligan) in ROM (a) Flexion ROM (b) Extension ROM; Figure S4: MET versus Subgroups of JM(non-Mulligan, 2-week Mulligan, 4-week Mulligan) in pain; Figure S5: MET versus Subgroups of JM(non-Mulligan, 2-week Mulligan, 4-week Mulligan) in disability; Figure S6: MET versus Subgroups of JM(non-Mulligan, 2-week Mulligan, 4-week Mulligan) in ROM (a) Flexion ROM (b) Extension ROM. Ref. [53] is cited in Supplementary File.

## Abbreviations

CI	Confidence interval.
CNFDS	Copenhagen Neck Functional Disability Scale.
EMBASE	Excerpta Medica database.
GRADE	Grading of Recommendations, Assessment, Development, and Evaluation.
JM	Joint mobilization.
MCID	Minimal clinically important difference.
MD	Mean difference.
MeSH	Medical Subject Headings.
MET	Muscle energy technique.
MNP	Mechanical neck pain.
NAGs	Natural apophyseal glides.
NDI	Neck Disability Index.
NPRS	Numeric Pain Rating Scale.
PICOS	Population, Intervention, Comparison, Outcome, Study design.
PRISMA	Preferred Reporting Items for Systematic Reviews and Meta-Analyses.
PROSPERO	International Prospective Register of Systematic Reviews.
RCT	Randomized controlled trial.
RevMan	Review Manager.
RI	Reciprocal inhibition.
RoB	Risk of bias
ROM	Range of motion
SMD	Standardized mean difference
SNAGs	Sustained natural apophyseal glides
VAS	Visual Analogue Scale
