# Supplementary material for: Head-to-Head Comparison of Muscle Energy Technique and Joint Mobilization on Pain Improvement in Mechanical Neck Pain and Related Outcomes: A Systematic Review and Meta-Analysis
Source: Healthcare (Basel). 2026 Nov 2;14(21):3719. doi: 10.3390/healthcare14213719 (PMC13763013; doi:10.3390/healthcare14213719)
Supplement: Supplementary file 1 [file healthcare-14-03719-s001.zip › healthcare-4562057-supplementary.pdf]

Supplementary Table S1. Search strategies

PubMed (38)	("Neck Pain"[MeSH Terms] OR "Neck Pain"[Title/Abstract] OR "cervical pain"[Title/Abstract] OR "non-specific neck pain"[Title/Abstract] OR "chronic neck pain"[Title/Abstract] OR "mechanical neck pain"[Title/Abstract])<br>AND ("manipulation; osteopathic"[MeSH Terms] OR "muscle energy technique"[Title/Abstract] OR “MET”[Title/Abstract] OR "post-isometric relaxation"[Title/Abstract] OR "reciprocal inhibition"[Title/Abstract])<br>AND ("Musculoskeletal Manipulations"[MeSH Terms] OR "mobilisation"[Title/Abstract] OR "mobilization"[Title/Abstract] OR "SNAG"[Title/Abstract] OR "sustained natural apophyseal glides"[Title/Abstract])<br>AND ("randomized controlled trial"[Publication Type] OR "randomized controlled trial"[Title/Abstract] OR "RCT"[Title/Abstract] OR "trial"[Title/Abstract] OR "comparative study"[Title/Abstract])
Cochrane library (41)	#1 MeSH descriptor: [Neck Pain] explode all trees<br>#2 "Neck pain" or "cervical pain"<br>#3 #1 or #2<br>#4 "Mobilization" or "mobilisation" or "SNAG" or "Sustained Natural Apophyseal Glide"<br>#5 MeSH descriptor: [Musculoskeletal Manipulations] explode all trees<br>#6 "Muscle Energy Technique" or "post isometric relaxation" or "autogenic inhibition"<br>#7 MeSH descriptor: [Manipulation, Osteopathic] explode all trees<br>#8 (#4 or #5) and (#6 or #7)<br>#9 "randomized controlled trial" OR "RCT" OR "comparative study"<br>#10 #3 and #8 and #9
EMBASE (45)	('neck pain'/exp OR 'cervical pain'/exp<br>OR 'non-specific neck pain':ti,ab,kw<br>OR 'chronic neck pain':ti,ab,kw<br>OR 'mechanical neck pain':ti,ab,kw<br>OR 'cervicalgia':ti,ab,kw)<br>AND<br>('muscle energy technique'/exp<br>OR 'muscle energy technique':ti,ab,kw<br>OR met:ti,ab,kw<br>OR 'post-isometric relaxation':ti,ab,kw<br>OR 'reciprocal inhibition':ti,ab,kw)<br>AND<br>('joint mobilization'/exp<br>OR 'mobiliz*':ti,ab,kw<br>OR 'mobilis*':ti,ab,kw<br>OR sNAG:ti,ab,kw<br>OR 'sustained natural apophyseal glides':ti,ab,kw)<br>AND<br>('randomized controlled trial'/exp<br>OR 'single blind procedure'/exp<br>OR 'double blind procedure'/exp<br>OR 'crossover procedure'/exp<br>OR random*:ti,ab,kw<br>OR placebo*:ti,ab,kw<br>OR 'prospective study'/exp)<br>NOT ('animal'/exp NOT 'human'/exp)

Supplementary Table S2. Excluded studies after full-text review

Sachdeva, 2019*	Use of McGill Pain Questionnaire, resulting in incompatibility for quantitative synthesis (meta-analysis)

*, Sachdeva S, Yadav J, Gulati M. Comparing the efficacy of first rib Maitland mobilisation and muscle energy technique on pain; disability and head position sense in patients with chronic mechanical neck pain. J Clin Diagn Res. 2019;13(12):YC11-YC19.

Supplementary Table S3. Data Extraction for Meta-Analysis

Author, year	Intervention	N	Pain		Disability		Flexion ROM (°)		Extension ROM (°)	
			Pre	Post	Pre	Post	Pre	Post	Pre	Post
Abbas, 2024[37]	MET + CT	20	6.35 ± 0.99†	1.15 ± 0.75†	15.55 ± 4.71	2.95 ± 0.99	61.00 ± 6.81	81.25 ± 3.19	54.50 ± 6.47	66.75 ± 2.93
	JM (Maitland) + CT	20	6.20 ± 1.12†	1.40 ± 0.88†	16.85 ± 4.27	3.25 ± 0.96	62.25 ± 9.38	80.00 ± 3.62	53.75 ± 4.83	66.25 ± 3.19
	CT	20	6.30 ± 1.13†	2.50 ± 0.76†	17.85 ± 3.41	6.95 ± 1.19	60.50 ± 6.67	72.50 ± 4.14	52.75 ± 4.99	59.75 ± 4.13
Jamil, 2022[33]	MET + CT	22	8.27 ± 0.45	4.00 ± 0.69	41.36 ± 2.27	20.00 ± 3.45	36.23 ± 3.71	44.68 ± 4.90	21.14 ± 6.15	42.95 ± 3.33
	JM (NAGs) + CT	23	8.26 ± 0.44	2.43± 0.50	41.30 ± 2.24	12.17 ± 2.53	35.74 ± 3.75	68.48 ± 4.87	21.30 ± 6.25	62.17 ± 5.99
Malik, 2025[36]	MET + CT	27	8.90 ± 1.09	6.90 ± 1.16	29.03 ± 3.07	19.13 ± 4.13	35.03 ± 3.03	57.07 ± 11.50	42.40 ± 4.36	62.77 ± 6.02
	JM (SNAGs) + CT	28	8.37 ± 1.13	2.30 ± 0.95	28.90 ± 2.99	8.20 ± 3.38	34.47 ± 2.90	66.93 ± 8.16	44.77 ± 2.98	83.90 ± 9.02
Sezerel, 2024[35]	MET + ET	25	5.69 ± 3.06	2.52 ± 2.53	14.44 ± 6.81	7.08 ± 5.01	NR	NR	NR	NR
	JM (Cyriax) + ET	26	5.69 ± 1.68	2.35 ± 1.68	14.50 ± 6.24	8.62 ± 6.40	NR	NR	NR	NR
	ET	25	5.12 ± 2.80	2.13 ± 2.02	12.92 ± 7.58	6.80 ± 6.58	NR	NR	NR	NR
Tabassum, 2023[34]	MET + CT	32	6.69 ± 1.21	2.89 ± 1.39	47.83 ± 9.97	10.37 ± 4.06	38.53 ± 3.79	58.51 ± 5.72	40.06 ± 3.43	50.43 ± 3.58
	JM (Maitland; SNAGs) + CT	34	6.51 ± 1.04	2.74 ± 1.04	43.06 ± 7.88	10.97 ± 2.77	39.89 ± 3.29	56.01 ± 6.95	39.80 ± 3.54	59.03 ± 4.26
	CT	33	6.46 ± 1.22	4.17 ± 1.15	44.06 ± 9.55	16.94 ± 2.48	39.57 ± 3.30	47.46 ± 3.58	40.09 ± 3.76	45.09 ± 3.06
Tank, 2018[32]	MET + CT	20	6.64 ± 1.26	3.00 ± 1.28	30.78 ± 9.09	12.72 ± 3.28	37.39 ± 6.10	43.94 ± 4.10	39.72 ± 5.20	50.72 ± 4.20
	JM (SNAGs) + CT	20	6.82 ± 0.75	2.40 ± 0.58	32.00 ± 6.56	13.47 ± 3.56	38.42 ± 6.80	47.00 ± 3.58	41.58 ± 4.60	53.42 ± 3.30

MET, Muscle Energy Technique; JM, Joint Mobilization; CT, Conventional Therapy; ET, Electro-Thermal Therapy; SNAGs, Sustained Natural Apophyseal Glides; NAGs, Natural Apophyseal Glides; NPRS, Visual Analog Scale; NDI, Neck Disability Index; CNFDS, Copenhagen Neck Functional Disability Scale; NR, Not Reported.

†, Pain scores were standardized to a 0–10 scale; VAS (0–100 mm) or NPRS (0-100) data were rescaled by dividing by 10 (higher scores indicate worse pain); ††, Disability was assessed using the NDI in most studies, whereas one study used the CNFDS.[33]

Supplementary Table S4. Intervention protocols of included studies

Author, year	Dose	MET	JM	CT
Abbas, 2024[36]	12 sessions (3×/wk, 4 wk)	Isometric contraction (20% strength); 7-10 s hold; 5 s rest; PIR 30 s; Application (anterior-middle-posterior scalenes, upper trapezius, levator scapulae, SCM); 3-5 repetitions.	Maitland mobilization (grades I-II/III-IV); 30 s duration; Application (C7-T1); Prone position; 3 repetitions.	Hot pack application (10 min, cervical-upper thoracic region); Dynamic neck exercises (flexion, extension, side bending, rotation); 8-10 repetitions; Isometric strengthening with TheraBand; 5-10 repetitions; 6 s hold.
Jamil, 2022[33]	16 sessions (4×/wk, 4 wk)	Isometric contraction (20% strength); 7-10 s hold; PIR 30 s; Application (upper trapezius, levator scapulae, SCM); Supine position.	Mulligan NAGs (glides toward eyeballs in facet plane); Application (cervical vertebrae for flexion); Sitting position; Oscillation (2-3/s); 5-10 s duration.	None
Malik, 2025[37]	12 sessions (3×/wk, 4 wk)	MET application; Experienced physiotherapists; Details not specified.	Mulligan SNAGs application; Experienced physiotherapists; Details not specified. application	Hot pack application; Active neck range-of-motion exercises; Details not specified.
Sezerel, 2024[35]	12 sessions (3×/wk, 4 wk)	Isometric contraction (20% strength); 7 s hold; PIR; Application (bilaterally anterior-middle-posterior scalenes, upper trapezius, levator scapulae, SCM); 3 repetitions.	Deep friction massage (3-4 min); Spasmic nuchal muscles; Cyriax's techniques (bridging, cervical manual traction, rotation with manual traction, anteroposterior glide with manual traction, lateral glide); Traction (2-3 s); 3-5 repetitions.	Hot pack application (20 min); Trapezius and suboccipital muscles; TENS (80 Hz, 70 µs-180 µs, 20 min); Therapeutic ultrasound (continuous mode, 1 MHz, 1.5 wt/cm², 10 min)
Tabassum, 2023[34]	6 sessions (3×/wk, 2 wk)	Isometric contraction (30-50% strength); 7-10 s hold; PIR 5 s; Application (anterior-middle-posterior scalenes, upper trapezius, levator scapulae, SCM); 3-5 repetitions.	Maitland mobilization (grades I-III); 15 unilateral postero-anterior glides (C2-C7); Extension Mulligan SNAGs; hypomobile segments; 5 repetitions.	Isometric exercises (2 sets, 5-10 repetitions); Serratus anterior, middle and lower trapezius, deep cervical flexors, rhomboids; Stretching (20 s, 5 repetitions); Pectoralis, suboccipital, levator scapulae, upper trapezius.
Tank, 2018[32]	6 sessions (3×/wk, 2 wk)	Isometric contraction (20% of available strength) held for 5 s; PIR for 5-7s; applied in supine to upper (C1-C2) and lower (C3-C7) cervical vertebrae; 2-3 repetitions.	Mulligan SNAGs (glides directed toward the eyeballs in the facet plane); applied in sitting to cervical vertebrae for flexion, extension, rotation, and lateral flexion; 6-10 repetitions.	Moist hot pack (15-20 min on neck region); Isometric neck exercises for flexion, extension, rotation, side-bending (10 s, 10-15 repetitions); Sitting position.

MET, Muscle Energy Technique; JM, Joint Mobilization; CT, Conventional Treatment; ET, Electro-Thermal Therapy; PIR, Post-isometric relaxation; SNAG, Sustained Natural Apophyseal Glide; CTJ, Cervicothoracic Junction; SCM, Sternocleidomastoid.

Supplementary Table S5. Sensitivity analysis of Pain intensity (leave-one-out method)

Author, year	Pooled estimate (MD of NPRS)	95% Confidence Interval		*p* value	I^2 (%)
		lower	upper		
Abbas, 2024[36]	1.44	-0.13	3.01	0.07	97
Jamil, 2022[33]	1.06	-0.87	3.00	0.28	98
Malik, 2025[37]	0.47	-0.34	1.29	0.25	90
Sezerel, 2024[35]	1.33	-0.23	2.90	0.09	98
Tabassum, 2023[34]	1.36	-0.29	3.00	0.11	98
Tank, 2018[32]	1.26	-0.42	2.95	0.14	98

Pain scores from VAS and NPRS were harmonized to a 0–10 scale (VAS means and SDs divided by 10 where reported in mm) and pooled using MDs. VAS, Visual Analogue Scale; NPRS, Numeric Pain Rating Scale; MD, mean difference.

Supplementary Table S6. Sensitivity analysis of Disability (leave-one-out method)

Author, year	Pooled estimate (SMD of NDI, CNFDS)	95% Confidence Interval		*p* value	I^2 (%)
		lower	upper		
Abbas, 2024[36]	0.92	-0.33	2.17	0.15	95
Jamil, 2022[33]	0.35	-0.63	1.34	0.48	93
Malik, 2025[37]	0.28	-0.57	1.14	0.52	90
Sezerel, 2024[35]	0.92	-0.36	2.20	0.16	95
Tabassum, 2023[34]	0.90	-0.42	2.22	0.18	95
Tank, 2018[32]	1.91	-0.35	2.17	0.16	95

NDI, Neck Disability Index; CNFDS, Copenhagen Neck Functional Disability Scale; SMD, standardized mean difference.

Supplementary Table S7. Sensitivity analysis of flexion ROM (leave-one-out method)

Author, year	Pooled estimate (MD of flexion ROM)	95% Confidence Interval		*p* value	I^2 (%)
		lower	upper		
Abbas, 2024[36]	8.54	-3.55	20.63	0.17	98
Jamil, 2022[33]	1.79	-0.22	5.79	0.38	87
Malik, 2025[37]	5.76	-5.29	16.82	0.31	99
Tabassum, 2023[34]	8.83	-2.67	20.34	0.13	99
Tank, 2018[32]	7.45	-5.60	20.50	0.26	99

ROM, cervical range of motion; MD, mean difference.

Supplementary Table S8. Sensitivity analysis of extension ROM (leave-one-out method)

Author, year	Pooled estimate (MD of extension ROM, º)	95% Confidence Interval		*p* value	I^2 (%)
		lower	upper		
Abbas, 2024[36]	12.80	5.00	20.59	0.001	97
Jamil, 2022[33]	7.79	0.77	14.80	0.03	97
Malik, 2025[37]	7.46	-0.08	15.00	0.05	98
Tabassum, 2023[34]	10.55	-0.19	20.90	0.05	98
Tank, 2018[32]	12.01	2.69	21.32	0.01	98

ROM, cervical range of motion; MD, mean difference.

Supplementary Table S9. Comparison of Pooled Estimates and Heterogeneity: Overall Analysis vs. 4-week Mulligan Subgroup

Outcome	Subgroup	Pooled estimate (MD or SMD)	95% Confidence Interval		*p* value	I^2 (%)
			lower	upper		
Pain	All included	1.15	-0.26	2.57	0.11	97
	4-wk Mulligan	3.08	0.11	6.05	0.04	99
Disability	All included	0.71	-0.34	1.76	0.18	94
	4-wk Mulligan	2.71	2.15	3.26	0.00001	0
Flexion	All included	6.55	-3.01	16.11	0.00001	98
	4-wk Mulligan	17.01	3.36	30.67	0.01	95
Extension	All included	10.11	2.69	17.53	0.008	98
	4-wk Mulligan	19.84	17.52	22.15	0.00001	0

Pain scores from VAS and NPRS were harmonized to a 0–10 scale (VAS means and SDs divided by 10 where reported in mm) and pooled using MDs. VAS, Visual Analogue Scale; NPRS, Numeric Pain Rating Scale; MD, mean difference; SMD, standardized mean difference; wk, week.

Supplementary Fig. S1. MET versus Subgroups of JM(non-Mulligan, Mulligan) in pain.

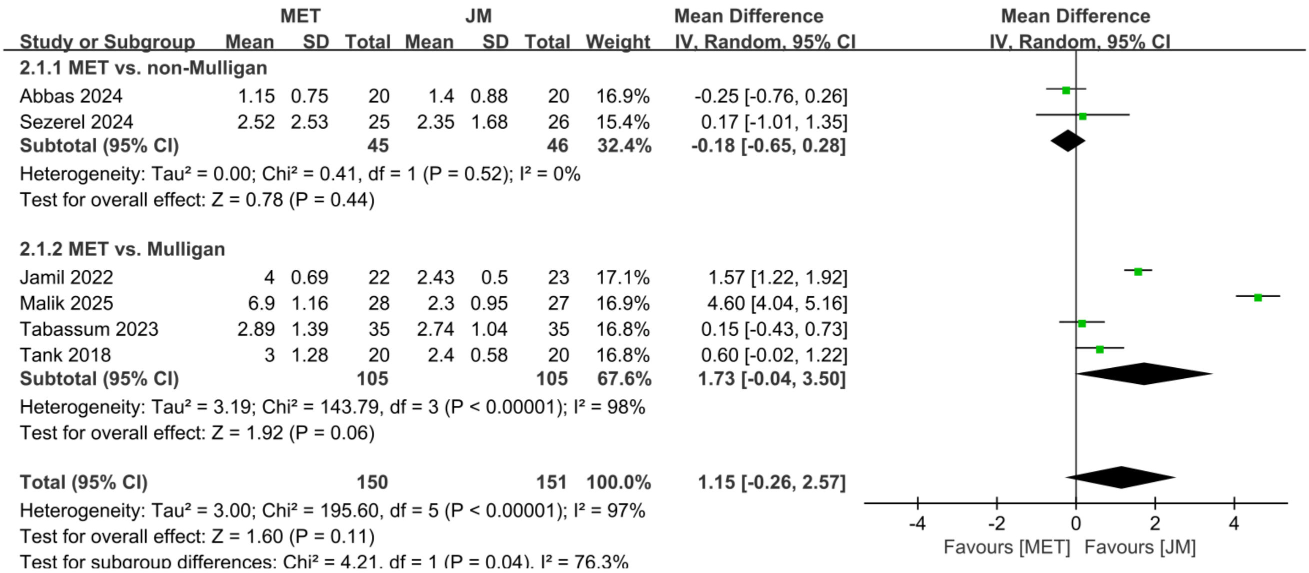

Pain scores from VAS and NPRS were harmonized to a 0–10 scale (VAS means and SDs divided by 10 where reported in mm) and pooled using MDs. MET, Muscle Energy Technique; JM, Joint Mobilization; VAS, Visual Analogue Scale; NPRS, Numeric Pain Rating Scale; MD, mean difference; SMD, standardized mean difference.

Supplementary Fig. S2. MET versus Subgroups of JM(non-Mulligan, Mulligan) in disability.

Study or Subgroup	MET Mean	MET SD	MET Total	JM Mean	JM SD	JM Total	Weight	Std. Mean Difference IV, Random, 95% CI
**5.1.1 MET vs. non-Mulligan**								
Abbas 2024	2.95	0.99	20	3.25	0.96	20	16.7%	-0.30 [-0.93, 0.32]
Sezerel 2024	7.08	5.01	25	8.62	6.4	26	17.0%	-0.26 [-0.81, 0.29]
**Subtotal (95% CI)**			**45**			**46**	**33.7%**	**-0.28 [-0.69, 0.13]**
Heterogeneity: Tau² = 0.00; Chi² = 0.01, df = 1 (P = 0.93); I² = 0%								
Test for overall effect: Z = 1.33 (P = 0.18)								
**5.1.2 MET vs. Mulligan**								
Jamil 2022	20	3.45	22	12.17	2.53	23	16.1%	2.55 [1.75, 3.36]
Malik 2025	19.13	4.13	28	8.2	3.38	27	16.3%	2.85 [2.09, 3.61]
Tabassum 2023	10.37	4.06	35	10.97	2.77	35	17.2%	-0.17 [-0.64, 0.30]
Tank 2018	12.72	3.28	20	13.47	3.56	20	16.7%	-0.21 [-0.84, 0.41]
**Subtotal (95% CI)**			**105**			**105**	**66.3%**	**1.23 [-0.35, 2.81]**
Heterogeneity: Tau² = 2.49; Chi² = 72.25, df = 3 (P < 0.00001); I² = 96%								
Test for overall effect: Z = 1.53 (P = 0.13)								
**Total (95% CI)**			**150**			**151**	**100.0%**	**0.71 [-0.34, 1.76]**
Heterogeneity: Tau² = 1.62; Chi² = 86.69, df = 5 (P < 0.00001); I² = 94%								
Test for overall effect: Z = 1.33 (P = 0.18)								
Test for subgroup differences: Chi² = 3.28, df = 1 (P = 0.07), I² = 69.5%								

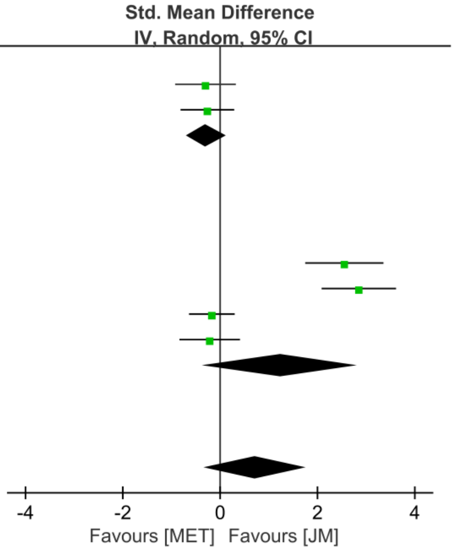

MET, Muscle Energy Technique; JM, Joint Mobilization; MD, mean difference; SMD, standardized mean difference; NDI, Neck Disability Index; CNFDS, Cervical Neck Functional Disability Scale.

Supplementary Fig. S3. MET versus Subgroups of JM(Mulligan) in ROM.

(a) Flexion ROM.

Study or Subgroup	MET Mean	MET SD	MET Total	JM Mean	JM SD	JM Total	Weight	Mean Difference IV, Random, 95% CI
Jamil 2022	-44.68	4.9	22	-68.48	4.87	23	25.2%	23.80 [20.94, 26.66]
Malik 2025	-57.07	11.5	28	-66.93	8.16	27	24.4%	9.86 [4.60, 15.12]
Tabassum 2023	-58.51	5.72	35	-56.01	6.95	35	25.2%	-2.50 [-5.48, 0.48]
Tank 2018	-43.94	4.1	20	-47	3.58	20	25.3%	3.06 [0.67, 5.45]
**Total (95% CI)**			**105**			**105**	**100.0%**	**8.54 [-3.55, 20.63]**
Heterogeneity: Tau² = 148.98; Chi² = 182.18, df = 3 (P < 0.00001); I² = 98%								
Test for overall effect: Z = 1.38 (P = 0.17)								

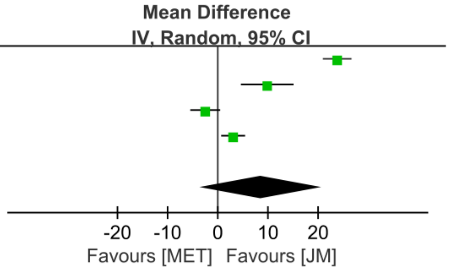

(b) Extension ROM.

Study or Subgroup	MET Mean	MET SD	MET Total	JM Mean	JM SD	JM Total	Weight	Mean Difference IV, Random, 95% CI
Jamil 2022	-42.95	3.33	22	-62.17	5.99	23	25.0%	19.22 [16.40, 22.04]
Malik 2025	-62.77	6.02	28	-83.9	9.02	27	24.2%	21.13 [17.06, 25.20]
Tabassum 2023	-50.43	3.58	35	-59.03	4.26	35	25.5%	8.60 [6.76, 10.44]
Tank 2018	-50.72	4.2	20	-53.42	3.3	20	25.3%	2.70 [0.36, 5.04]
**Total (95% CI)**			**105**			**105**	**100.0%**	**12.80 [5.00, 20.59]**
Heterogeneity: Tau² = 61.17; Chi² = 109.47, df = 3 (P < 0.00001); I² = 97%								
Test for overall effect: Z = 3.22 (P = 0.001)								

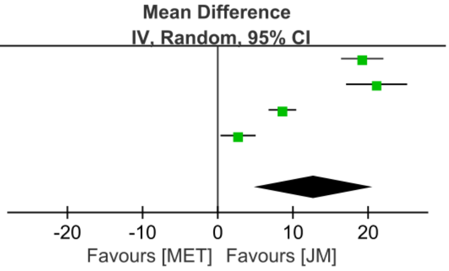

MET, Muscle Energy Technique; JM, Joint Mobilization; MD, mean difference; SMD, standardized mean difference; ROM, Range of Motion.

Supplementary Fig. S4. MET versus Subgroups of JM(non-Mulligan, 2-wk Mulligan, 4-wk Mulligan) in pain.

Study or Subgroup	MET Mean	MET SD	MET Total	JM Mean	JM SD	JM Total	Weight	Mean Difference IV, Random, 95% CI
**3.1.1 MET vs. non-Mulligan**								
Abbas 2024	1.15	0.75	20	1.4	0.88	20	16.9%	-0.25 [-0.76, 0.26]
Sezerel 2024	2.52	2.53	25	2.35	1.68	26	15.4%	0.17 [-1.01, 1.35]
**Subtotal (95% CI)**			**45**			**46**	**32.4%**	**-0.18 [-0.65, 0.28]**
Heterogeneity: Tau² = 0.00; Chi² = 0.41, df = 1 (P = 0.52); I² = 0%								
Test for overall effect: Z = 0.78 (P = 0.44)								
**3.1.2 MET vs. 2-wk Mulligan**								
Tabassum 2023	2.89	1.39	35	2.74	1.04	35	16.8%	0.15 [-0.43, 0.73]
Tank 2018	3	1.28	20	2.4	0.58	20	16.8%	0.60 [-0.02, 1.22]
**Subtotal (95% CI)**			**55**			**55**	**33.6%**	**0.36 [-0.08, 0.80]**
Heterogeneity: Tau² = 0.01; Chi² = 1.10, df = 1 (P = 0.30); I² = 9%								
Test for overall effect: Z = 1.61 (P = 0.11)								
**3.1.3 MET vs. 4-wk Mulligan**								
Jamil 2022	4	0.69	22	2.43	0.5	23	17.1%	1.57 [1.22, 1.92]
Malik 2025	6.9	1.16	28	2.3	0.95	27	16.9%	4.60 [4.04, 5.16]
**Subtotal (95% CI)**			**50**			**50**	**34.0%**	**3.08 [0.11, 6.05]**
Heterogeneity: Tau² = 4.53; Chi² = 80.54, df = 1 (P < 0.00001); I² = 99%								
Test for overall effect: Z = 2.03 (P = 0.04)								
**Total (95% CI)**			**150**			**151**	**100.0%**	**1.15 [-0.26, 2.57]**
Heterogeneity: Tau² = 3.00; Chi² = 195.60, df = 5 (P < 0.00001); I² = 97%								
Test for overall effect: Z = 1.60 (P = 0.11)								
Test for subgroup differences: Chi² = 6.60, df = 2 (P = 0.04), I² = 69.7%								

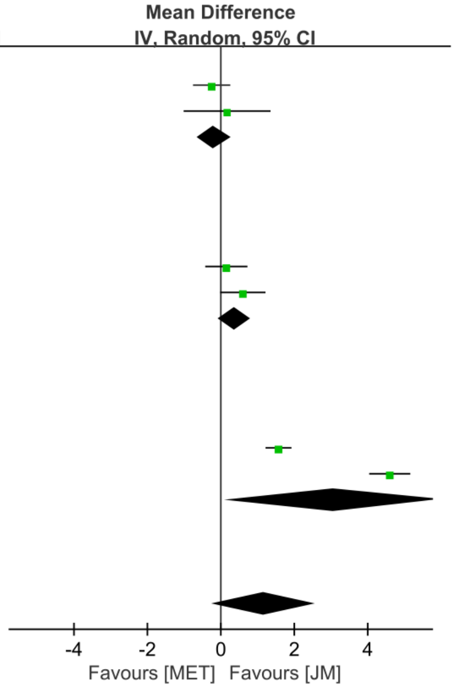

Pain scores from VAS and NPRS were harmonized to a 0–10 scale (VAS means and SDs divided by 10 where reported in mm) and pooled using mean differences (MDs). MET, muscle energy technique; JM, joint mobilization; VAS, Visual Analogue Scale; NPRS, Numeric Pain Rating Scale; MD, mean difference; SMD, standardized mean difference.

Supplementary Fig. S5. MET versus Subgroups of JM(non-Mulligan, 2-wk Mulligan, 4-wk Mulligan) in disability.

Study or Subgroup	MET Mean	MET SD	MET Total	JM Mean	JM SD	JM Total	Weight	Std. Mean Difference IV, Random, 95% CI
**6.1.1 MET vs. non-Mulligan**								
Abbas 2024	2.95	0.99	20	3.25	0.96	20	16.7%	-0.30 [-0.93, 0.32]
Sezerel 2024	7.08	5.01	25	8.62	6.4	26	17.0%	-0.26 [-0.81, 0.29]
**Subtotal (95% CI)**			**45**			**46**	**33.7%**	**-0.28 [-0.69, 0.13]**
Heterogeneity: Tau² = 0.00; Chi² = 0.01, df = 1 (P = 0.93); I² = 0%								
Test for overall effect: Z = 1.33 (P = 0.18)								
**6.1.2 MET vs. 2-wk Mulligan**								
Tabassum 2023	10.37	4.06	35	10.97	2.77	35	17.2%	-0.17 [-0.64, 0.30]
Tank 2018	12.72	3.28	20	13.47	3.56	20	16.7%	-0.21 [-0.84, 0.41]
**Subtotal (95% CI)**			**55**			**55**	**33.9%**	**-0.19 [-0.56, 0.19]**
Heterogeneity: Tau² = 0.00; Chi² = 0.01, df = 1 (P = 0.91); I² = 0%								
Test for overall effect: Z = 0.98 (P = 0.33)								
**6.1.3 MET vs. 4-wk Mulligan**								
Jamil 2022	20	3.45	22	12.17	2.53	23	16.1%	2.55 [1.75, 3.36]
Malik 2025	19.13	4.13	28	8.2	3.38	27	16.3%	2.85 [2.09, 3.61]
**Subtotal (95% CI)**			**50**			**50**	**32.4%**	**2.71 [2.15, 3.26]**
Heterogeneity: Tau² = 0.00; Chi² = 0.28, df = 1 (P = 0.60); I² = 0%								
Test for overall effect: Z = 9.58 (P < 0.00001)								
**Total (95% CI)**			**150**			**151**	**100.0%**	**0.71 [-0.34, 1.76]**
Heterogeneity: Tau² = 1.62; Chi² = 86.69, df = 5 (P < 0.00001); I² = 94%								
Test for overall effect: Z = 1.33 (P = 0.18)								
Test for subgroup differences: Chi² = 86.39, df = 2 (P < 0.00001), I² = 97.7%								

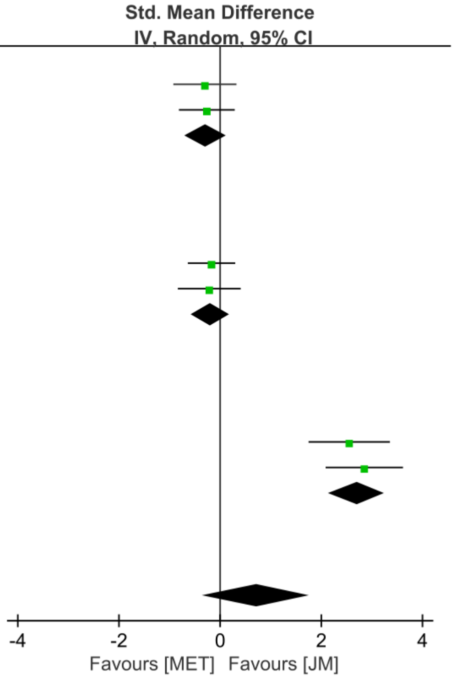

MET, Muscle Energy Technique; JM, Joint Mobilization; MD, mean difference; SMD, standardized mean difference; NDI, Neck Disability Index; CNFDS, Cervical Neck Functional Disability Scale.

Supplementary Fig. S6. MET versus Subgroups of JM(non-Mulligan, 2-wk Mulligan, 4-wk Mulligan) in ROM.

(a) Flexion ROM.

Study or Subgroup	MET Mean	MET SD	MET Total	JM Mean	JM SD	JM Total	Weight	Mean Difference IV, Random, 95% CI
**9.1.1 MET vs. 2-wk Mulligan**								
Tabassum 2023	-58.51	5.72	35	-56.01	6.95	35	25.2%	-2.50 [-5.48, 0.48]
Tank 2018	-43.94	4.1	20	-47	3.58	20	25.3%	3.06 [0.67, 5.45]
**Subtotal (95% CI)**			**55**			**55**	**50.4%**	**0.35 [-5.09, 5.80]**
Heterogeneity: Tau² = 13.56; Chi² = 8.14, df = 1 (P = 0.004); I² = 88%								
Test for overall effect: Z = 0.13 (P = 0.90)								
**9.1.2 MET vs. 4-wk Mulligan**								
Jamil 2022	-44.68	4.9	22	-68.48	4.87	23	25.2%	23.80 [20.94, 26.66]
Malik 2025	-57.07	11.5	28	-66.93	8.16	27	24.4%	9.86 [4.60, 15.12]
**Subtotal (95% CI)**			**50**			**50**	**49.6%**	**17.01 [3.36, 30.67]**
Heterogeneity: Tau² = 92.51; Chi² = 20.87, df = 1 (P < 0.00001); I² = 95%								
Test for overall effect: Z = 2.44 (P = 0.01)								
**Total (95% CI)**			**105**			**105**	**100.0%**	**8.54 [-3.55, 20.63]**
Heterogeneity: Tau² = 148.98; Chi² = 182.18, df = 3 (P < 0.00001); I² = 98%								
Test for overall effect: Z = 1.38 (P = 0.17)								
Test for subgroup differences: Chi² = 4.93, df = 1 (P = 0.03), I² = 79.7%								

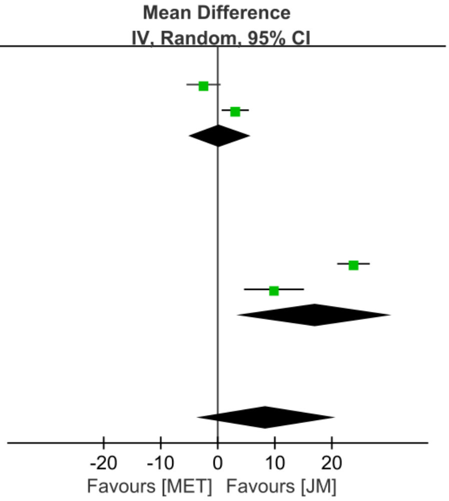

(b) Extension ROM.

Study or Subgroup	MET Mean	MET SD	MET Total	JM Mean	JM SD	JM Total	Weight	Mean Difference IV, Random, 95% CI
**12.1.1 MET vs. 2-wk Mulligan**								
Tabassum 2023	-50.43	3.58	35	-59.03	4.26	35	25.5%	8.60 [6.76, 10.44]
Tank 2018	-50.72	4.2	20	-53.42	3.3	20	25.3%	2.70 [0.36, 5.04]
**Subtotal (95% CI)**			**55**			**55**	**50.8%**	**5.70 [-0.09, 11.48]**
Heterogeneity: Tau² = 16.25; Chi² = 15.06, df = 1 (P = 0.0001); I² = 93%								
Test for overall effect: Z = 1.93 (P = 0.05)								
**12.1.2 MET vs. 4-wk Mulligan**								
Jamil 2022	-42.95	3.33	22	-62.17	5.99	23	25.0%	19.22 [16.40, 22.04]
Malik 2025	-62.77	6.02	28	-83.9	9.02	27	24.2%	21.13 [17.06, 25.20]
**Subtotal (95% CI)**			**50**			**50**	**49.2%**	**19.84 [17.52, 22.15]**
Heterogeneity: Tau² = 0.00; Chi² = 0.57, df = 1 (P = 0.45); I² = 0%								
Test for overall effect: Z = 16.79 (P < 0.00001)								
**Total (95% CI)**			**105**			**105**	**100.0%**	**12.80 [5.00, 20.59]**
Heterogeneity: Tau² = 61.17; Chi² = 109.47, df = 3 (P < 0.00001); I² = 97%								
Test for overall effect: Z = 3.22 (P = 0.001)								
Test for subgroup differences: Chi² = 19.81, df = 1 (P < 0.00001), I² = 95.0%								

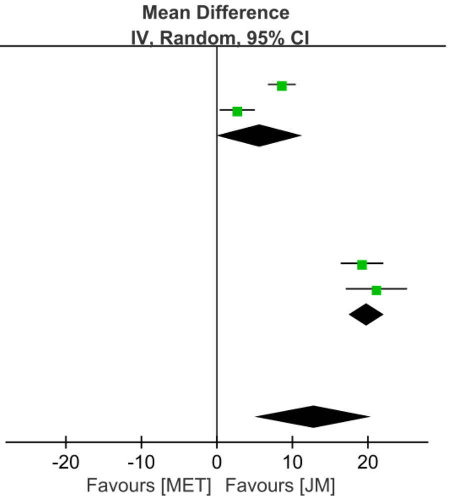

MET, Muscle Energy Technique; JM, Joint Mobilization; MD, mean difference; SMD, standardized mean difference; ROM, Range of motion.
